# Aggressive Digital Papillary Adenocarcinoma Mimicking a Giant Cell Tumour – A Case Report and Review of the Literature

**DOI:** 10.7759/cureus.9531

**Published:** 2020-08-03

**Authors:** Kenneth Joyce, Niamh Leonard, Christoph Theopold

**Affiliations:** 1 Plastic, Reconstructive, and Aesthetic Surgery, Mater Hospital, Dublin, IRL; 2 Plastic and Reconstructive Surgery, Mater Hospital, Dublin, IRL; 3 Histopathology, St. James' Hospital, Dublin, IRL; 4 Plastic and Reconstructive Surgery, St. James' Hospital, Dublin, IRL

**Keywords:** aggressive digital papillary adenocarcinoma, sentinel lymph node biopsy, eccrine adnexal tumour

## Abstract

Aggressive digital papillary adenocarcinoma (ADPAca) is a rare, underreported, and often misdiagnosed malignant tumour of the eccrine sweat gland, with high recurrence and metastatic potential. We present a case of a painless mass over the middle phalanx of the dominant index finger in a 51-year-old man. The mass was present for over 20 years, which had slowly increased in size. The patient only presented when it began to interfere with his profession as an electrician. The clinical presentation was consistent with a giant cell tumour. Histological diagnosis was of an ADPAca. Staging investigations were negative and he subsequently went on to have a ray amputation. The importance of high clinical suspicion of digit lesions is highlighted. No specific histologic features have been identified to predict recurrence or metastasis. We review the merits of performing sentinel node biopsy and alternative treatment options such as Moh’s micrographic surgery. We review the international literature to assess metastatic potential and follow-up requirements.

## Introduction

Aggressive digital papillary adenocarcinoma (ADPAca) is a rare, underreported, malignant tumour of the eccrine sweat gland, with high recurrence and metastatic potential [1]. No specific histologic features have been identified to predict recurrence or metastasis [1]. The classical presentation of this neoplasm is a single mass, almost exclusively on the fingers or toes [2]. Given the rarity of this malignancy, ADPAca is often misdiagnosed as a pyogenic granuloma, angioma, mucoid cyst or multiple other benign growths. We present the case of a patient with a painless, slow-growing mass that clinically resembled a giant cell tumour. We review the international literature and discuss the current controversies in the management of ADPAca, the extent of surgical resection required, and the merits of performing a sentinel lymph node biopsy.

## Case presentation

**Figure 1:**
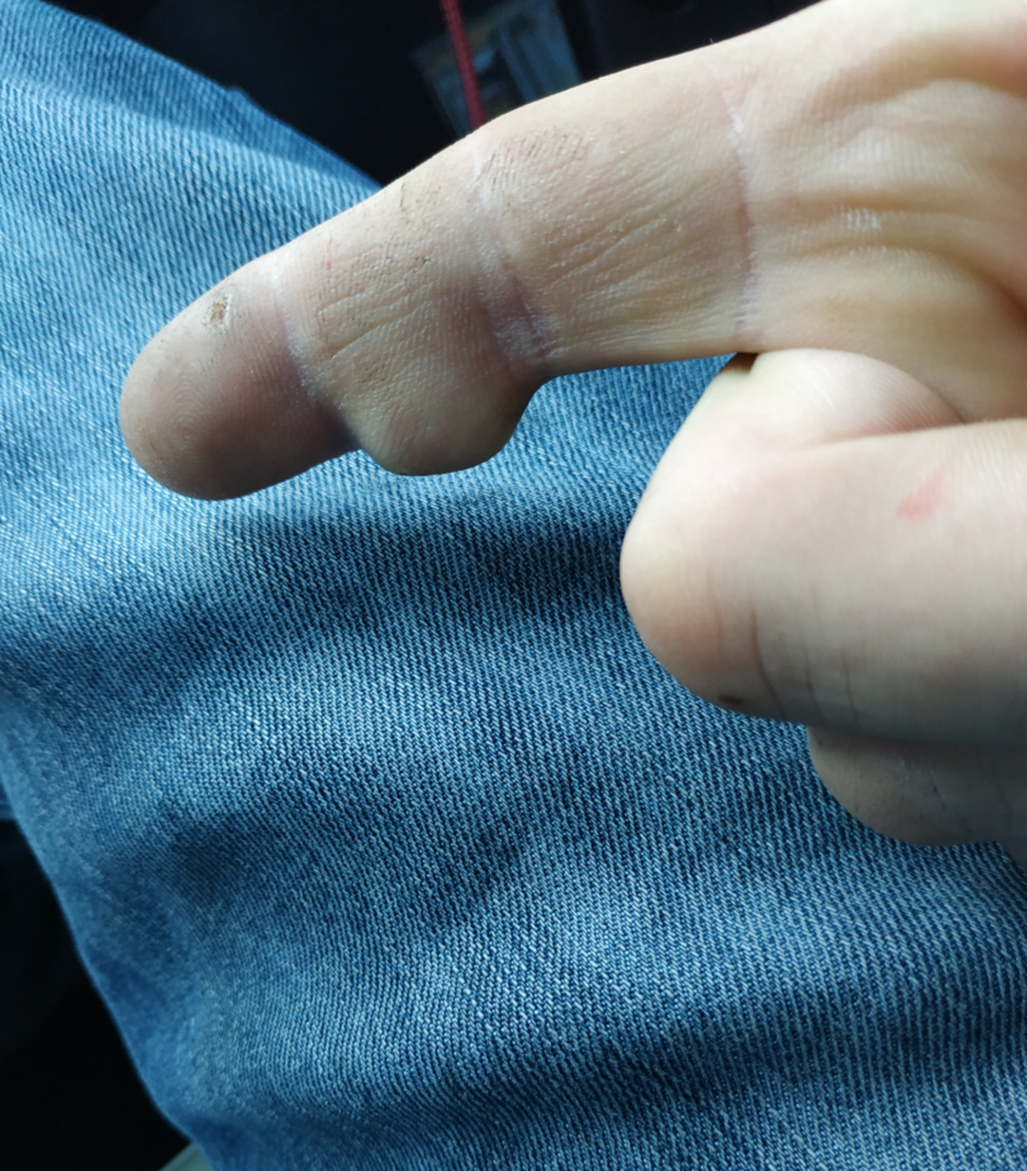
Pre-operative clinical photo 1

**Figure 2:**
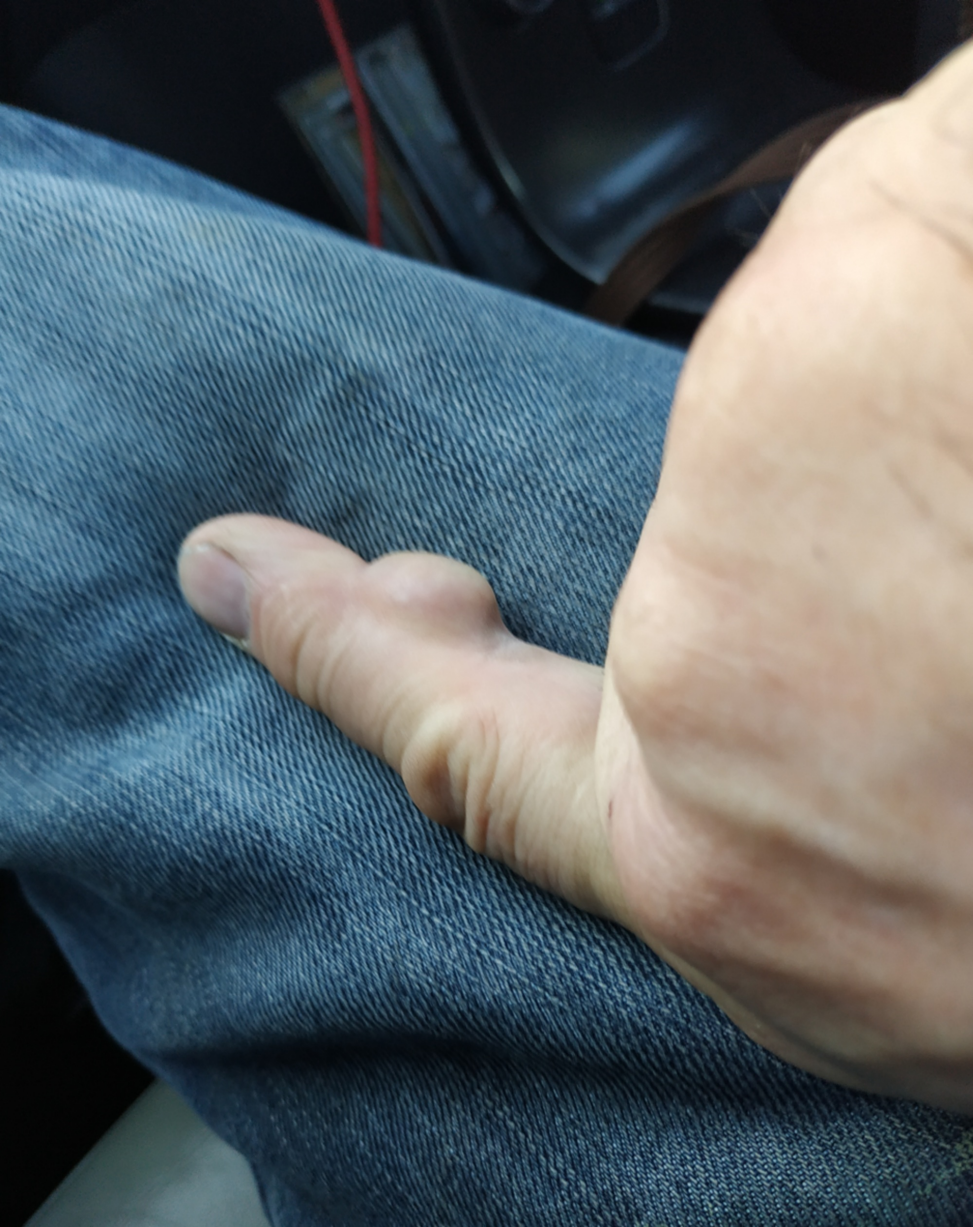
Pre-operative clinical photo 2

A 51-year-old, right-hand-dominant electrician presented to the outpatient clinic, with a slow-growing, painless mass over the middle phalanx of his dominant index finger (Figures 1-2).

**Figure 3:**
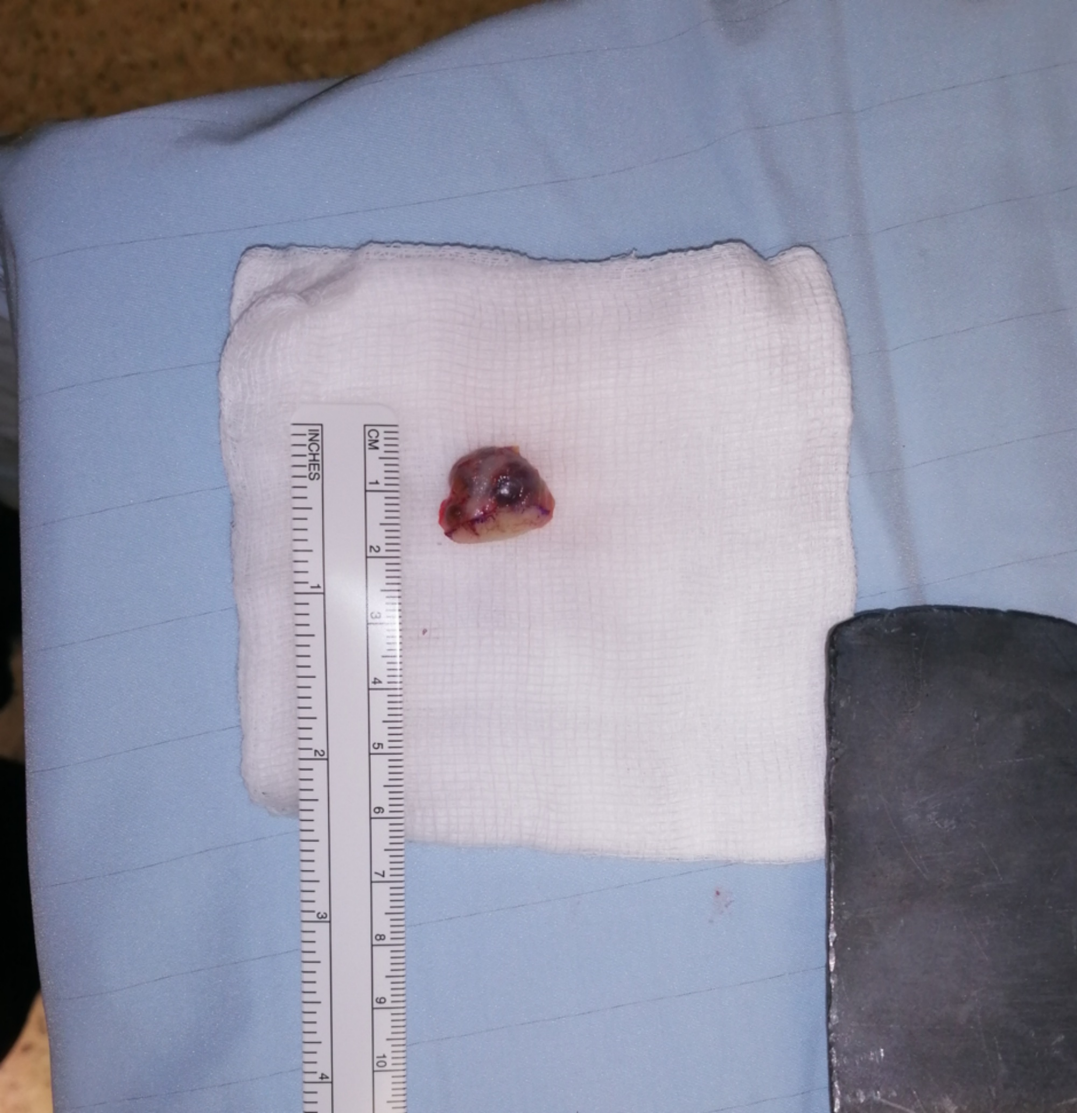
Excisional biopsy specimen

This had been present for over 20 years. The size of the mass was beginning to interfere with his working duties; however, there was no neurosensory deficit. He had no significant past medical history. Clinically, the lesion was consistent with a giant cell tumour. He had no axillary lymphadenopathy. A close-margin excision biopsy of the mass with some overlying skin (Figure 3) showed a dermal tumour that was lobular and partially cystic.

**Figure 4:**
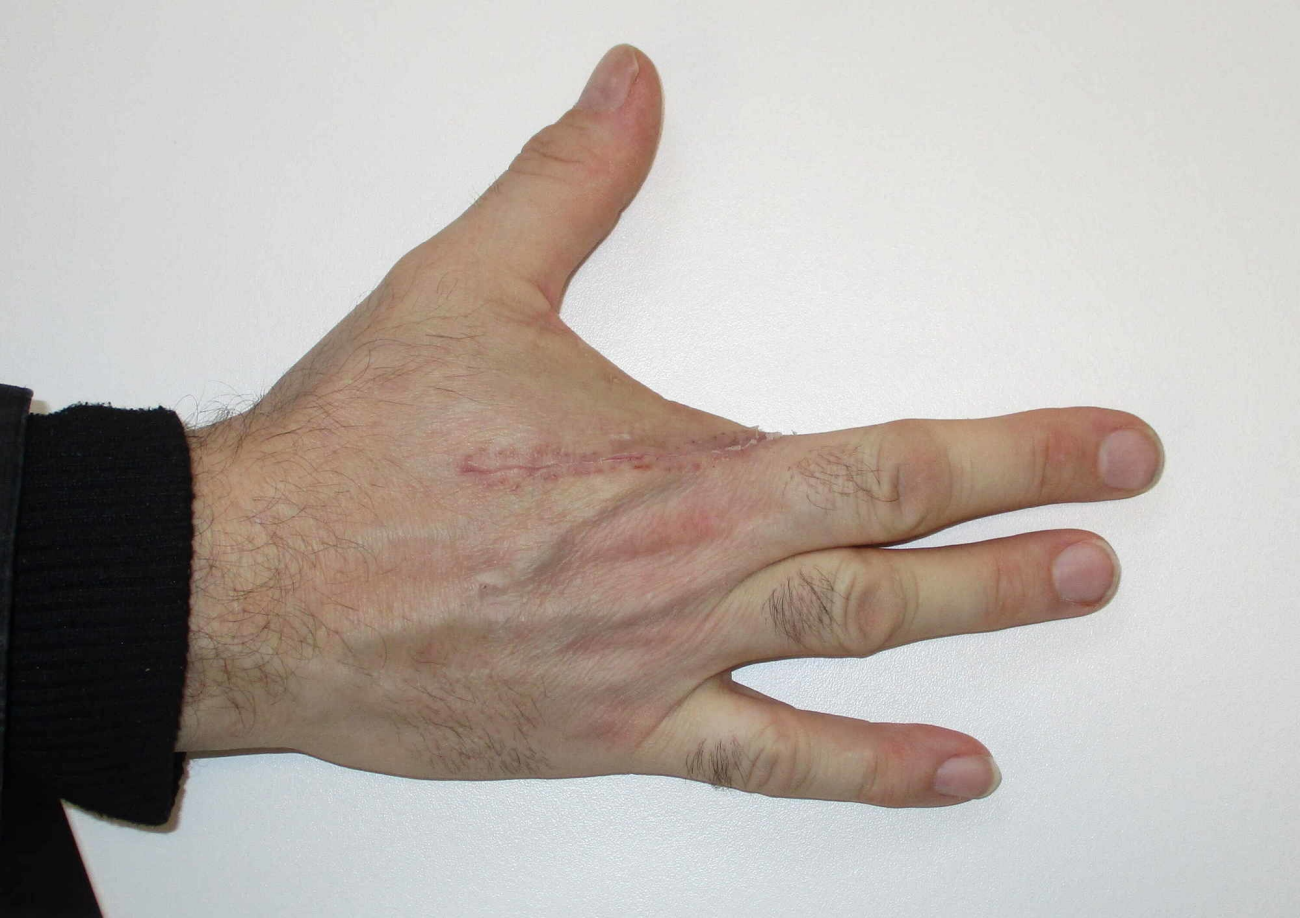
Post-operative clinical photograph

There were tubulopapillary structures with large irregular nuclei and numerous mitoses. It was diagnosed as an ADPAca. A staging positron emission tomography-computed tomography (PET-CT) scan was negative for distant metastases. Following a discussion at our institution's skin cancer multidisciplinary team meeting, it was concluded that a sentinel node biopsy was not warranted, and he subsequently went on to have a ray amputation of his index finger (Figure 4).

**Figure 5:**
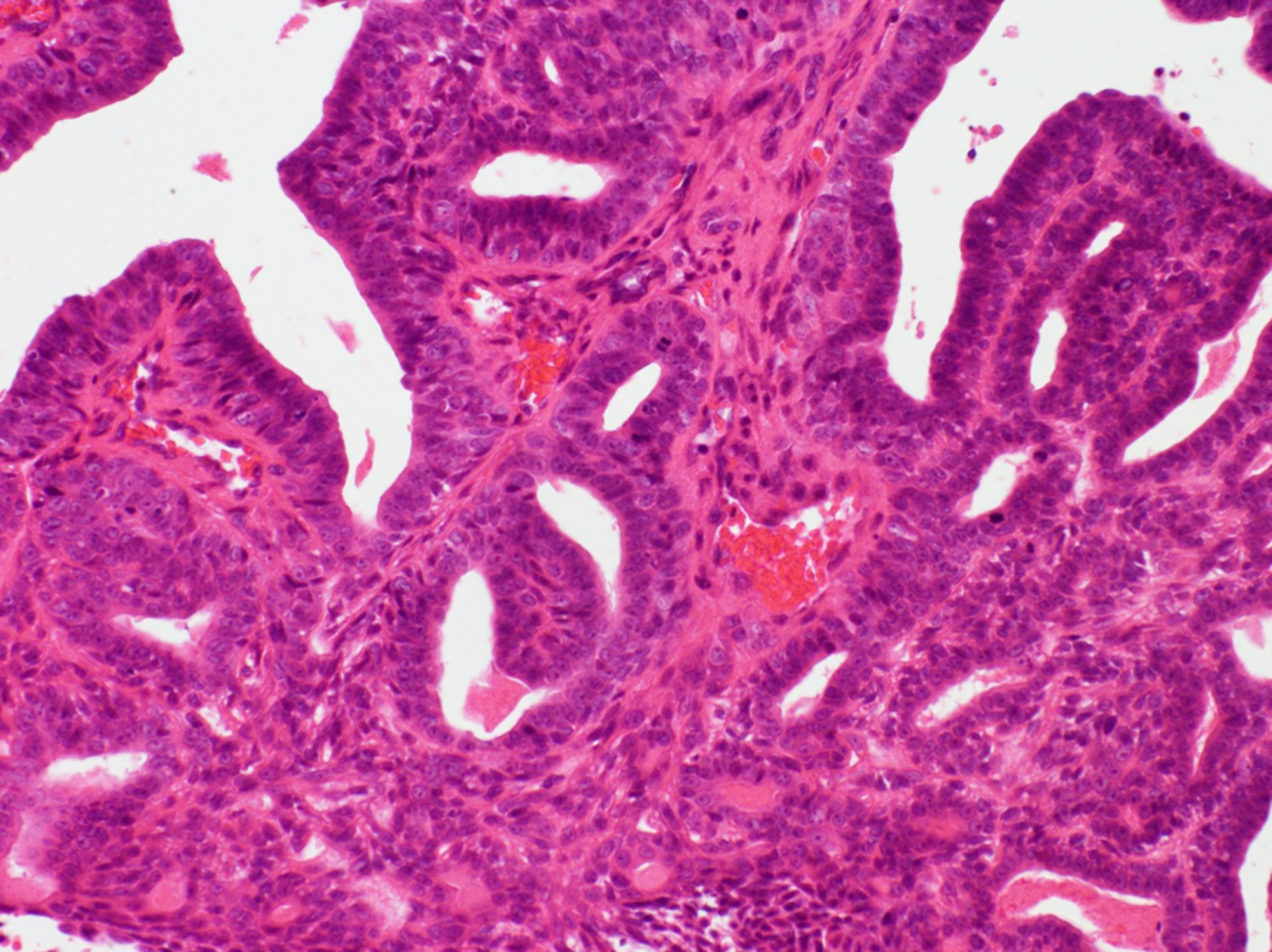
High magnification histological image showing an adenocarcinoma forming tubular and papillary structures aligned by atypical, hyperchromatic cells with mitoses H&E stain x200 magnification H&E: haemotoxylin and eosin

**Figure 6:**
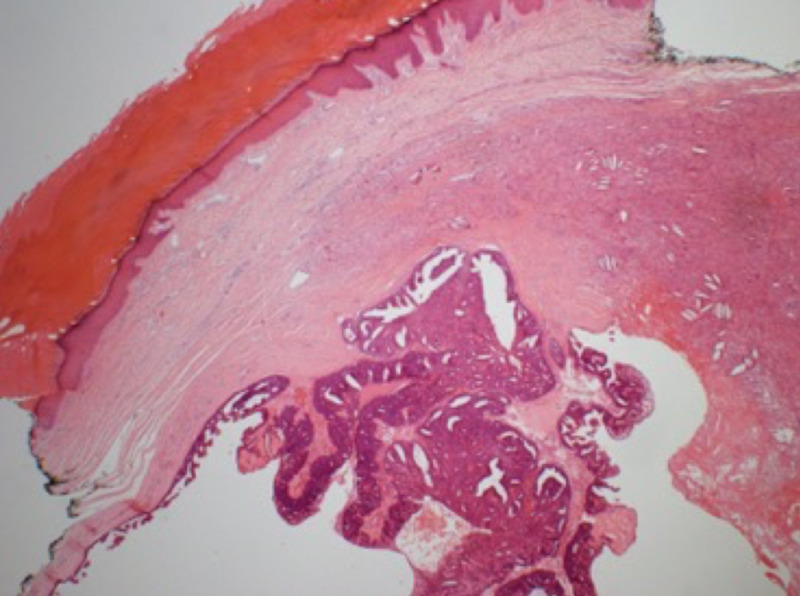
The tumour is dermal and partially cystic with intraluminal papillary projections H&E stain x20 magnification H&E: haemotoxylin and eosin

He had an uncomplicated post-operative course. Final histology showed involvement of the skin only, with the lesion widely excised (Figures 5-6).

## Discussion

Aggressive digital papillary adenocarcinoma was first described by Helwig in 1979. It is a malignant adnexal tumour presenting as a nodule on the volar surface of the digits of the hands and, less commonly, of the feet, with an incidence of 0.08 per million person-years [1]. Characteristic histologic features, including tubuloalveolar and ductal structures with areas of papillary projections protruding into the cystic lumina. In 1987, Kao et al. published a large case series of 57 patients and defined criteria to distinguish papillary digital adenomas from adenocarcinomas [2]. Early studies suggested that poor glandular differentiation, cellular atypia, and pleomorphism as pathological features of adenocarcinoma [2]. However, further studies of this dataset showed a high rate of local recurrence and metastasis of digital adenomas [3]. Subsequent conclusions were that all lesions suspected to be adenomas should be treated as ADPAca, as it is currently difficult to distinguish histologically between the two pathologies [3].

Presentation and diagnosis

The neoplasm typically occurs as a single, painless mass, almost exclusively on the fingers, toes, and adjacent skin of the palms and soles [2]. They most commonly occur in males in their fifties to seventies. These are frequently poorly circumscribed, involving the dermis and subcutis. Most are grossly cystic and have a predilection to occur in digits, most commonly the distal phalanx. A cutaneous biopsy is required for histological diagnosis. This painless mass can mimic a pyogenic granuloma, angioma, mucoid cyst, giant cell tumour, wart, felon, or foreign body granuloma. Pathological differential diagnoses include benign adnexal tumours such as hidradenoma, papillary eccrine adenoma, apocrine hidrocystoma, and metastatic papillary adenocarcinoma. This can often result in a delay in diagnosis and treatment.

Sentinel lymph node biopsy

The feasibility, accuracy, and validity of sentinel lymph node biopsy (SLNB) in staging the regional nodes of patients is well defined for certain skin tumours, e.g. melanoma. Its role in the staging of ADPAca is, however, poorly defined. It was first described in ADPAca in 2000 by Malafa et al. [4]. The true incidence of SLNB positivity is difficult to determine due to the paucity of patients in small series.

Table 1 demonstrates the largest series published in the international literature with regard to SLNB in ADPAca. There is variation between units, compounded by the low number published overall.

**Table 1:** Largest international series of SLNB and metastasis in ADPAca NR = not recorded; SLNB: SLNB; ADPAca: aggressive digital papillary adenocarcinoma [2,4-9]

Study	Year	No. of patients	SLNB performed (positive)	Local recurrence (%)	Rate of metastasis (%)
Kao [2]	1987	17	0/17	8/17 (47%)	7/17 (41%)
Malafa [4]	2000	1	1/1 (1/1)	NR	NR
Bogner [5]	2003	2	2/2 (1/2)	0/2 (0%)	0/2 (0%)
Weingertner [6]	2017	19	2/19 (0/2)	4/17 (24%)	1/17 (6%)
Suchak [7]	2012	31	4/31 (0/4)	5/23 (22%)	6/23 (26%)
Kempton [8]	2015	1	1/1 (1/1)	NR	NR
Yokota [9]	2018	2	2/2 (0/2)	0/2 (0%)	0/2 (0%)

Sentinel lymph node biopsy may identify metastasis earlier than would otherwise be detected clinically, but the survival benefit of this is undetermined [5]. Head and neck oncologists often cite a threshold for performing SLNB as a 20% or greater chance of positive nodes for mucosal tumours [10]. A threshold of a 5% chance of having positive nodes is often used for skin melanomas [4]. A population based-study based on Surveillance, Epidemiology, and End Results (SEER) data by Rismiller et al. has determined the rate of regional disease spread to be 22.3% [1]. Given the high rate of metastasis in ADPAca, with significant involvement of regional nodes, many units will offer SLNB to these patients [6].

ADPAca tumours appear to behave similarly to melanoma in their predilection to metastasize. The role of sentinel node biopsy as a determinant of prognosis and the potential survival impact of regional lymphadenectomy remains to be determined. Elective regional node dissection for skin tumours, such as melanoma, is poorly accepted for routine staging because of the morbidity associated with nodal dissections. Since there is currently no effective chemotherapy for ADPAca, some authors would argue that performing staging procedures, such as SLNB, is of little benefit [11]. A sentinel node with micrometastasis or low tumour burden leads to a clinical dilemma about the merits of performing a completion lymphadenectomy without any proven survival benefit.

Treatment

The treatment of ADPAca has evolved as knowledge about this lesion has increased. Current recommendations for the management of ADPAca are to control the disease locally by wide excision of the tumour or digit amputation, with long-term follow-up for recurrence or metastatic spread [1]. Some authors have advocated conservative excision, sparing the involved digit. In Cribier’s series of 19 cases, conservative surgical treatment, performed in seven of 19 cases, did not result in more recurrences than amputation [6]. However, Duke et al. report a 5% recurrence rate in patients with amputation or re-excision after their initial excision as compared with 50% recurrence in those who did not undergo subsequent re-excision or amputation [3]. There has been one case report of using Mohs micrographic surgery as a digit-sparing surgical approach to ADPAca [12]. Authors cite the indication for this technique in scenarios where the tumour is limited to the dermis, without extension into deeper anatomic structures [12].

Follow-up and outcomes

Metastasis occurs in approximately 15 to 30 per cent of cases [3]. Suchak et al. published a series of 31 cases in 2012, concluding that histologic features were not found to be predictive of outcome [7]. Therefore, these tumours should all be considered low-grade malignant tumours with no benign counterpart. Kao et al. reported a local recurrence rate of 48% and a metastasis rate of 41%, with 70% of metastases occurring in the lung [2]. These authors recommend a yearly clinical exam and chest X-rays for 10 years as a follow-up.

There is no good evidence supporting the use of adjuvant chemotherapy or radiotherapy in ADPAca. This is often reserved for palliative treatment. Surowy et al. have recently carried out transcriptome profiling on ADPAca and identified the overexpression of fibroblast growth factor FGFR2 [13]. Other sequencing analysis showed BRAF V600E mutations in one out of nine tumours [14]. Future chemotherapeutic targets will likely require molecular characterization of recurrent and metastatic ADPAca samples [13].

## Conclusions

ADPAca remains a rare, yet aggressive, cancer with an often benign presentation and insidious growth. Considering this neoplasm as a differential diagnosis in digital masses is important because of the potential for aggressive local growth and distant metastasis. No specific histologic features have been identified to predict recurrence or metastasis. Given the rarity of ADPAca, studies are limited by small sample size. Future directions will require the combination of independent data samples for significant conclusions. The role of sentinel node biopsy continues to be debated. Advances in chemotherapeutic profiling may promote the role of sentinel node biopsy as a prognostic tool.
